# Evergene: an interactive webtool for large-scale gene-centric analysis of primary tumours

**DOI:** 10.1093/bioadv/vbae092

**Published:** 2024-06-18

**Authors:** Anna Kennedy, Ella Richardson, Jonathan Higham, Panagiotis Kotsantis, Richard Mort, Barbara Bo-Ju Shih

**Affiliations:** Division of Biomedical and Life Sciences, Faculty of Health and Medicine, Lancaster University, Lancaster LA1 4YG, United Kingdom; Division of Biomedical and Life Sciences, Faculty of Health and Medicine, Lancaster University, Lancaster LA1 4YG, United Kingdom; Department of Mathematics and Statistics, Faculty of Science and Technology, Lancaster University, Lancaster LA1 4YF, United Kingdom; Division of Biomedical and Life Sciences, Faculty of Health and Medicine, Lancaster University, Lancaster LA1 4YG, United Kingdom; Division of Biomedical and Life Sciences, Faculty of Health and Medicine, Lancaster University, Lancaster LA1 4YG, United Kingdom; Division of Biomedical and Life Sciences, Faculty of Health and Medicine, Lancaster University, Lancaster LA1 4YG, United Kingdom

## Abstract

**Motivation:**

The data sharing of large comprehensive cancer research projects, such as The Cancer Genome Atlas (TCGA), has improved the availability of high-quality data to research labs around the world. However, due to the volume and inherent complexity of high-throughput omics data, analysis of this is limited by the capacity for performing data processing through programming languages such as R or Python. Existing webtools lack functionality that supports large-scale analysis; typically, users can only input one gene, or a gene list condensed into a gene set, instead of individual gene-level analysis. Furthermore, analysis results are usually displayed without other sample-level molecular or clinical annotations. To address these gaps in the existing webtools, we have developed Evergene using R and Shiny.

**Results:**

Evergene is a user-friendly webtool that utilizes RNA-sequencing data, alongside other sample and clinical annotation, for large-scale gene-centric analysis, including principal component analysis (PCA), survival analysis (SA), and correlation analysis (CA). Moreover, Evergene achieves in-depth analysis of cancer transcriptomic data which can be explored through dimensional reduction methods, relating gene expression with clinical events or other sample information, such as ethnicity, histological classification, and molecular indices. Lastly, users can upload custom data to Evergene for analysis.

**Availability and implementation:**

Evergene webtool is available at https://bshihlab.shinyapps.io/evergene/. The source code and example user input dataset are available at https://github.com/bshihlab/evergene.

## 1 Introduction

In the era of open science where large cancer datasets are made publicly available *via* projects like The Cancer Genome Atlas (TCGA) Program (Chang *et al.* 2013), researchers can freely access valuable high-quality data, boosting their research capacity (Mangul *et al.* 2019). Complementary to this, several projects deliver pre-processed data from these publicly available databases through webtools that enable data visualization, further improving the accessibility to these resources for those lacking bioinformatics expertise (Cerami *et al.* 2012, Metsalu and Vilo 2015, Tang *et al.* 2019, Dwivedi *et al.* 2022).

When exploring gene expression in cancer datasets, principal component analysis (PCA) and survival analysis (SA) are two of the most commonly-used analysis methods. PCA is an unsupervised method for reducing the dimensionality of a dataset while retaining information on the overall variation (Hotelling 1933). SA encompasses a collection of methods used to analyse differential occurrence for specific events (such as death) between groups or for a given continuous variable; this has been applied to cancer samples with low or high expression of specific genes to infer their potential importance in cancers. Currently, there is no webtool available that provides integration between PCA, SA, and comprehensive sample annotations, such as all clinical survival outcomes, histology classification, race, age, and gender. Further, available molecular classifications from several TCGA pan-cancer projects, such as immune subtypes (Thorsson *et al.* 2018), molecular subtypes (Hoadley *et al.* 2018), and stemness index (Malta *et al.* 2018), are not so far integrated into existing webtools. Integration of these additional annotations would allow better stratification of results and provide further insight into gene expression patterns or clinical survival outcomes.

For survival outcome analytics, overall survival (OS) is a commonly-used endpoint and has the advantage of having minimal ambiguity for defining an OS event, as the event is registered as either alive or dead (Tolaney *et al.* 2021). However, OS does not distinguish non-cancer causes of death, nor does it necessarily reflect tumour aggressiveness. The use of other observed events, such as progression-free interval (PFI), disease-specific survival (DSS), and disease-free interval (DFI), has the advantage of shorter minimum follow-up time and potentially closer representation of the tumour biology (Tolaney *et al.* 2021). Existing webtools that implemented TCGA data for SA are usually based on OS; existing tools have allowed for a limited number of clinical outcomes as registered events in SA. Moreover, Idogawa *et al.* (2021) have identified issues with the handling of survival data in several of the existing webtools. To address these, we incorporate OS, PFI, DSS, and DFI data from the standardized dataset published by the TCGA pan-cancer clinical data resource (TCGA-CDR) (Liu *et al.* 2018).

With the ever-increasing volume of data, there is a growing interest in high-throughput analysis. The number of gene queries is another limiting factor with existing TCGA data webtools, which are predominately designed for single-gene analysis. While some of the webtools for interrogating TCGA data have a multi-gene mode, they are limited in functionality. Even when multiple gene input is available, the plots and statistics tables often need to be individually downloaded and the plot data are often not available to the users, who may wish to analyse them further.

To address these issues, we have developed a webtool, Evergene, that supports integrated PCA and SA with multi-gene input capacity. Evergene incorporates OS, PFI, DSS, and DFI data from the standardized dataset published by TCGA-CDR (Liu *et al.* 2018).

## 2 Methods

### 2.1 Data processing

R (version 4.3.1) and RStudio (version 2023.06) were used for processing data. Harmonized TCGA RNA-sequencing (RNA-seq) data (Thorsson *et al.* 2018) and associated clinical information were downloaded from TCGA database through the R package TCGAbiolink (Colaprico *et al.* 2015) (version 2.28.3; downloaded on 27 July 2023). The harmonized TCGA RNA-seq data have been mapped to the human reference genome GRCh38 by Genomic Data Commons (v37.0). TCGA projects with 80 or more primary tumour samples were selected for downstream analysis. R package SummarizedExperiment was used for extracting the downloaded data; transcript per million (TPM) values were used for indicating gene expression levels in graphical outputs, and unstranded count values were used for PCA.

A total of 27 TCGA projects, each reflecting a cancer type or subtype, were found to have more than 80 samples (*n* = 80–1111) (Supplementary Table S1). Based on the TCGA-CDR recommendations, PFI is suitable to serve as an endpoint for clinical survival outcomes for all but 1 (Pheochromocytoma and Paraganglioma, PCPG) of the 27 TCGA projects, whereas OS is recommended to be used with caution for four of these projects (BRCA, LGG, PRAD, and READ). Therefore, PFI was chosen as the default option for SA in Evergene. As none of the endpoint measures were recommended for PCPG, the PCPG project was excluded, making a total of 26 TCGA projects included in Evergene.

PCA was performed independently for each cancer project with all samples and all genes detected in more than 20% of the samples. R package EdgeR (Robinson *et al.* 2009) (version 3.42.4) was used for performing TMM normalization in log-normalized count per million (CPM) values were used for PCA through R-base function prcomp using gene IDs as columns. PCA loadings for each gene were determined using the R package factoextra (version 1.0.7) for estimating the strength of contribution for each gene to each PC. The top 20 PCs were selected for visualization. The top 20 contributing genes for each PC were selected based on the strength of contribution irrespective of the direction, and the colour display indicates the multiplication between the gene contribution to an individual PC as well as the PC to the overall contribution data; a darker colour indicates a stronger contribution.

Correlation analyses were calculated with R base function corr using the Pearson correlation option. SA was performed using R base package survival (version 3.5–5) using one of the four possible clinical survival outcome, including OS, PFI, DSS, and DFI. Details of these four clinical survival outcomes were taken from the TCGA-CDR (Liu *et al.* 2018).

Additional sample annotation and clinical survival outcomes were derived from Liu *et al.* (2018). Stemness indexes, mRNAsi (based on mRNA expression), and EREG-mRNAsi (epigenetically regulated mRNAsi; based on both mRNA expression and DNA methylation), are derived from Malta *et al.* (2018), who utilized machine learning methods to identify stemness features associated with oncogenic dedifferentiation. Immune subtypes and molecular subtypes are derived from Thorsson *et al.* (2018) and Hoadley *et al.* (2018). Several annotations were shortened or combined for clearer visualization within Evergene (Supplementary Table S2). Values were treated as not available (NA) if missing or labelled as ‘not evaluated’, ‘unknown’, ‘discrepancy’, ‘not applicable’, or ‘not evaluated’.

### 2.2 Setup of the web platform

The source code for Evergene is written in the R programming language, and the interactive ShinyApp web-application was developed using the R base package shiny (version 1.7.4.1). Several additional R packages were used to customize the user interface, including shinythemes (version 1.2.0), shinyWidgets (version 0.7.6), and shinyBS (version 0.61.1). The final web platform was hosted on shinyapps.io and extensively tested on multiple operating systems (Linux, Mac, Windows) and web-browsers (Chrome, Firefox, Safari, Microsoft Edge). PCA and correlation plots were made with R package ggplot2 (version 3.4.2) and plotly (version 4.10.2), and coloured with R package viridis (version 0.6.4). The full source code for the Evergene ShinyApp and associated data pre-processing can be found on Github https://github.com/bshihlab/evergene.

## 3 Results

### 3.1 Overview of Evergene

**Figure 1.**
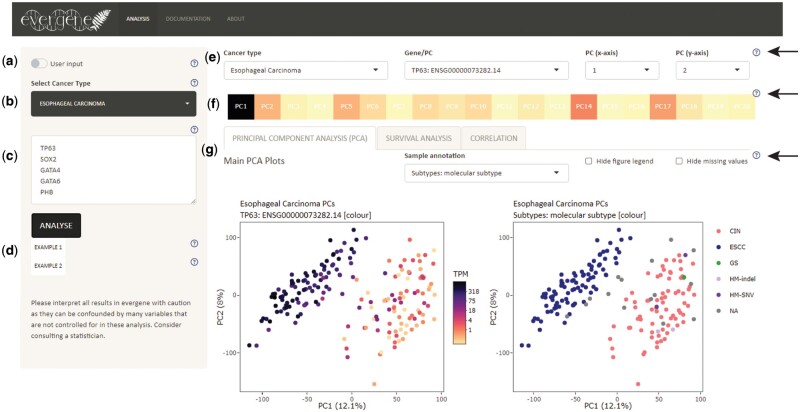
Overview of Evergene. This screenshot shows the output page for ‘Example 1’ shown in (d) on Evergene (TP63, SOX2, GATA4, GATA6, and PHB for input gene and Esophageal Carcinoma for input cancer). Arrows indicate question marks that can be used to bring up help messages. (a) The switch toggles on options for users to upload their own data for PCA and CA. (b) Alternatively, cancer projects from TCGA can be selected. (c) Genes of interest can be inputted before using the ‘analyse’ button to submit inputs for analysis. (d) Example inputs are available for both cancer projects and user inputs, which can be downloaded from https://github.com/bshihlab/evergene. (e) The top panel is used to switch between the displayed outputs for different cancer projects, genes, and PCs. (f) The middle panel indicates the strength of contribution for the currently selected gene towards each PC. This can be used to identify the potential PCs of interest for a given gene. (g) The bottom panel is comprised of three tabs and each contains the results for a different type of analysis.

Evergene is available at https://bshihlab.shinyapps.io/evergene. Within the webtool, users can provide their own data (Fig. 1a) or select from a list of TCGA cancer projects in addition to providing a list of input genes (Fig. 1b and c). For analysis using TCGA data, the gene inputs may be Ensembl gene IDs or Human Genome Organisation Gene Nomenclature Committee (HGNC) gene symbols. The maximum number of input gene–cancer combinations is limited to 100 due to computing constraints. This can be a combination of 10 genes in 10 cancers, or 100 genes in one cancer. Two example inputs are available, and each section contains explanatory notes under the question mark icons (Fig. 1d).

Three types of analyses are available in Evergene: PCA, SA, and CA. In the top panel, users can specify cancer projects and genes, along with two of the top 20 principal components (PCs) that they would like to display as the *x* and *y* axes in the PCA and SA plots (Fig. 1b). An overview of the contribution of the selected gene in each PC is indicated in the top panel (Fig. 1c). In the bottom panel (Fig. 1d), users can use the tab menu to switch between the results for PCA, SA, and CA.

Each analysis tab contains several plots with adjustable graphical inputs and buttons for downloading all plots and data. The main plots are Kaplan–Meier survival curve plots for SA, PCA scatter plots for the first 20 PCs, and scatter plots of selected variables for CA. Plots and data export are limited to the first 30 *y*-axis variables for CA and the first 100 gene-cancer combinations for SA and PCA.

### 3.2 Comparison to similar existing webtools

The two commonly-used PCA webtools, ClustVis (Metsalu and Vilo 2015) and GEPIA2 (Tang *et al.* 2019), both have different fundamental designs to Evergene (Table 1 and Supplementary Fig. S1). To our best knowledge, Evergene is the only webtool that performs whole-transcriptome PCA using TCGA data, as well as being the only webtool that annotates gene expression over PCA plots. ClustVis (Metsalu and Vilo 2015) can be used to perform PCA based on user input data or studies from the Array Express, but does not provide a list of cancer datasets. While GEPIA2 (Tang *et al.* 2019) has preloaded TCGA datasets, its PCA is performed on an input gene list, as opposed to Evergene which uses the full transcriptome. Unlike Evergene and ClustVis, GEPIA2 does not provide information on gene contribution towards each PC. Although ClustVis exports comprehensive PCA outputs as tables, it is not designed for interactive visualization and users need to plot graphs with external software. Lastly, PCA in Evergene is integrated with SA and CA, allowing users to explore potential relationships between PCs and other sample characteristics, such as survival, patient metadata, and molecular indices.

**Table 1.** Comparison between Evergene and existing webtools for principal component analysis (PCA).

Functionality	Evergene	GEPIA2	ClustVis
User input	Yes	No	Yes
Preloaded data: number of projects^a^; grouped cancer types	26; 26	33; 33	Studies of interest can be imported from Array Express
PCA method	Whole transcriptome	Selected genes^b^	Whole transcriptome, selected genes
Gene expression visualization	Yes	No	No
Sample annotation	Molecular (4), Clinical (up to 7)	Sample group (1)	Imported
Multiple plot export	Yes	No	No
Plot data export	Yes	No	Yes
Top contributing genes for each PC	Yes	No	Yes
Mix and match across multiple cancer/control studies	No	Yes	No
Gene contribution to each PC	Plot and data	No	Data
Number of principal components	Top 20	Top 10	Top 2 (plotted); all (data)
Interactive plots	Switch PCs. Sample annotation. Sample ID on hover	Switch PCs. Plot rotation (3D plot)	No

a Projects refers to cancer projects from which the data are originated from.

b Accepts up to approximately 250 genes; whole transcriptome cannot be selected.

There are several existing tools for SA, including Kaplan–Meier plotter [KM plotter] (Lánczky and Győrffy 2021), cBioPortal (Cerami *et al.* 2012, Gao *et al.* 2013, de Bruijn *et al.* 2023), Survival Genie (Dwivedi *et al.* 2022), GEPIA2 (Tang *et al.* 2019), and OncoLnc (Anaya 2016). The SA in Evergene has been compared and summarized in Supplementary Table S3 and Fig. S1. KM plotter is currently the only other webtool that accepts user input data; Survival Genie, GEPIA2, and OncoLnc are based on pre-loaded data. With the exception of GEPIA2 which has heatmap summaries on SA, these webtools are not designed for large numbers of individual gene queries; users need to perform and export the analysis on a gene-by-gene basis (Supplementary Table S3). Of the existing webtools, cBioPortal is the only one that shows comprehensive sample annotation for the groups of samples defined by high-/low-gene expression levels. Unlike cBioPortal, which shows summarized group information, Evergene plots sample annotation information for individual samples alongside SA and PCA.

Altogether, Evergene has the unique feature of integrating PCA, SA, and CA that allows for exploratory analysis on TCGA data with respect to their candidate genes, with interpretation aided by comprehensive sample metadata. Furthermore, Evergene is the only webtool which enables mass graph export when users have a large number of candidate genes or cancers of interest. Lastly, Evergene accepts in custom input data in the form of text files, allowing users to utilize all the above benefits with their own datasets. Example workflows are described in the Supplementary Fig. S2.

### 3.3 Case usage

**Figure 2.**
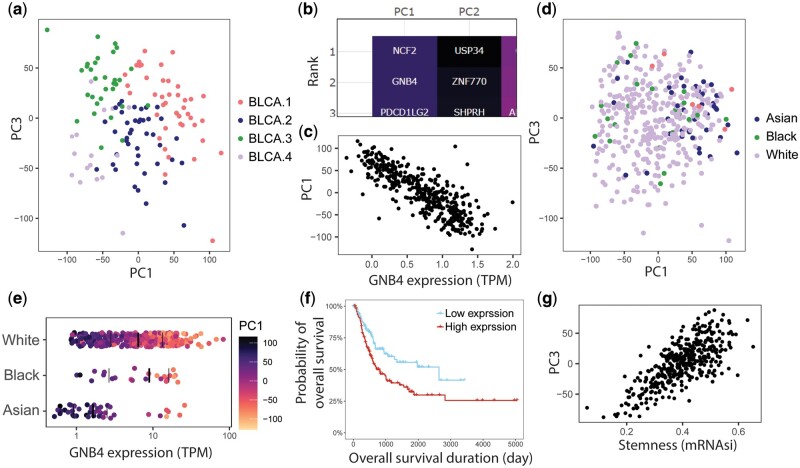
Case usage: identification of key genes that can be used to explain variations seen in bladder urothelial carcinoma. (a) PC1 and PC3 separate bladder urothelial carcinoma into molecular subtypes indicated by Hoadley *et al.* (2018). (b) In PC1, the second-highest contributing gene is GNB4, (c) which shows a strong negative correlation with PC1 (*r* = −0.83, *P* = 6.5E−104). (d) Most individuals with Asian ancestry are on the right-hand side for the PC1 axis, akin to those of BLCA.1 in (a). Thus, (e) Asian individuals are more likely to have low GNB4 expression, a grouping that is associated with higher probability of overall survival (*P* = .003 for log-rank test comparing top 33% to bottom 33%). (g) On the other hand, stemness index is strongly positively correlated with PC3 (*r* = 0.73, *P* = 1.8E−68).

For bladder urothelial carcinoma, the molecular subtypes BLCA.1–4, as defined by Hoadley *et al.* (2018), are separated by PC1 and PC3 (Fig. 2). Metascape pathway analysis has been performed using the top 100 genes contributing to PC1, which is significantly enriched in pathways related to inflammatory response (GO: 0006954. *P* < 1e−10) and regulation of immune cell activation (e.g. GO: 0050865 and GO: 0032944 for lymphocytes and mononuclear cells. *P* < 1e−10). Elevated systemic immune-inflammation index has been associated with poor survival outcomes for urothelial carcinoma (Liu *et al.* 2023). The second-highest contributing gene to PC1 is GNB4 (Fig. 2b), as shown in the final graph in the PCA tab. GNB4 is strongly correlated with PC1 (*r* = −0.83, *P* = 6.5E−104) and has higher expression in the BLCA.3 and BLCA.4, subtypes that are less common in those of Asian ancestry (Fig. 2d and e). Furthermore, the SA suggests higher GNB4 to be significantly (*P* < .01) associated with poorer progression-free interval, OS (Fig. 2f; *P* = .003 for log-rank test comparing top 33% to bottom 33%) and disease-specific survival; this is consistent with reports that elevated GNB4 protein levels is associated with poor prognosis in urothelial carcinoma (Chen *et al.* 2021). These observations suggest a potential difference in predisposition to inflammation-associated molecular subtypes in different ethnicities. On the other hand, PC3 is strongly correlated to the stemness index (mRNAsi) (Fig. 2g).

## 4 Limitations and future direction

There are functionalities available in other webtools that are not available in Evergene. For example, ClustVis can import Array Express datasets, Survival Genie correlates gene expression with immune cell compositions, and GEPIA2 can be used to find the genes with the highest differential survival (i.e. high expresser and low expresser have large difference in survival outcomes) for each cancer project as well as having summarized heatmap for SA. KM plotter, GEPIA2, and Survival Genie have additional parameters for restricting analysis to certain subsets of tumours or patient cohort. Evergene has restricted datasets to primary tumours; while this is to limit data variation to improve data stratification, some variations may be under-represented. Lastly, due to considerations around user accessibility and computational costs, most data processing steps are fixed, sample metadata does not include all available metadata from TCGA, and accepted inputs are restricted to 100 gene-cancer combinations. These areas can be further expanded in the future to reflect user needs.

## 5 Conclusion

In summary, Evergene provides a platform for gene-centric investigation of transcriptomic data across 26 cancer projects from TCGA, enabling the gene expression to be studied alongside clinical annotation and molecular classifications. The tool is designed for exploratory analysis, thus equipped with functionalities that enable a large number of gene-cancer inputs and download of many outputs. We have demonstrated the utility of Evergene in identifying potential biomarkers for histological subtypes and molecular subtypes that may reflect survival outcomes, such as TP63 and HID1 for differentiating squamous cell carcinoma from adenocarcinoma in multiple cancer types. The unsupervised analysis methods (PCA) in Evergene provide an overview of the transcriptomic variation across samples within cancer projects, and will therefore support the development of new molecular classification. We envisage Evergene will be a valuable exploratory tool for lay scientists in assisting the discovery of biomarkers in cancer.

## Supplementary Material

vbae092_Supplementary_Data

## Data Availability

The data underlying raw data used this article are available through the Genomic Data Commons Data Portal, at https://portal.gdc.cancer.gov.

## References

[vbae092-B1] Anaya J. OncoLnc: linking TCGA survival data to mRNAs, miRNAs, and lncRNAs. PeerJ Prepr 2016;4:e1780.

[vbae092-B2] Cerami E , Gao J, Dogrusoz U et al The cBio cancer genomics portal: an open platform for exploring multidimensional cancer genomics data. Cancer Discov 2012;2:401–4. 22588877 10.1158/2159-8290.CD-12-0095 PMC3956037

[vbae092-B3] Cancer Genome Atlas Research Network; Weinstein JN, Collisson EA et al The Cancer Genome Atlas Pan-Cancer analysis project. Nat Genet 2013;45:1113–20. 24071849 10.1038/ng.2764 PMC3919969

[vbae092-B4] Chen T-J , Dehghanian SZ, Chan T-C et al High G protein subunit beta 4 protein level is correlated to poor prognosis of urothelial carcinoma. Med Mol Morphol 2021;54:356–67. 34398348 10.1007/s00795-021-00301-w

[vbae092-B5] Colaprico A , Silva TC, Olsen C et al TCGAbiolinks: an R/Bioconductor package for integrative analysis of TCGA data. Nucleic Acids Res 2015;44:e71. 26704973 10.1093/nar/gkv1507 PMC4856967

[vbae092-B6] de Bruijn I , Kundra R, Mastrogiacomo B et al Analysis and visualization of longitudinal genomic and clinical data from the AACR project GENIE biopharma collaborative in cBioPortal. Cancer Res 2023;83:3861–7. 37668528 10.1158/0008-5472.CAN-23-0816 PMC10690089

[vbae092-B7] Dwivedi B , Mumme H, Satpathy S et al Survival Genie, a web platform for survival analysis across pediatric and adult cancers. Sci Rep 2022;12:3069. 35197510 10.1038/s41598-022-06841-0 PMC8866543

[vbae092-B8] Gao J , Aksoy BA, Dogrusoz U et al Integrative analysis of complex cancer genomics and clinical profiles using the cBioPortal. Sci Signal 2013;6:pl1. 23550210 10.1126/scisignal.2004088 PMC4160307

[vbae092-B9] Hoadley KA , Yau C, Hinoue T et al Cell-of-origin patterns dominate the molecular classification of 10,000 tumors from 33 types of cancer. Cell 2018;173:291–304.e296. 29625048 10.1016/j.cell.2018.03.022 PMC5957518

[vbae092-B10] Hotelling H. Analysis of a complex of statistical variables into principal components. J Educ Psychol 1933;24:417–41.

[vbae092-B151] Idogawa M , Koizumi M, Hirano T et al Dead or alive? Pitfall of survival analysis with TCGA datasets. Cancer Biol Ther 2021;22:527–8. 34530682 10.1080/15384047.2021.1979845 PMC8726696

[vbae092-B11] Lánczky A , Győrffy B. Web-based survival analysis tool tailored for medical research (KMplot): development and implementation. J Med Internet Res 2021;23:e27633. 34309564 10.2196/27633 PMC8367126

[vbae092-B12] Liu J , Lichtenberg T, Hoadley KA et al An integrated TCGA pan-cancer clinical data resource to drive high-quality survival outcome analytics. Cell 2018;173:400–16 e411. 29625055 10.1016/j.cell.2018.02.052 PMC6066282

[vbae092-B13] Liu J , Wu P, Lai S et al Prognostic models for upper urinary tract urothelial carcinoma patients after radical nephroureterectomy based on a novel systemic immune-inflammation score with machine learning. BMC Cancer 2023;23:574. 37349696 10.1186/s12885-023-11058-z PMC10286456

[vbae092-B14] Malta TM , Sokolov A, Gentles AJ et al Machine learning identifies stemness features associated with oncogenic dedifferentiation. Cell 2018;173:338–54.e315. 29625051 10.1016/j.cell.2018.03.034 PMC5902191

[vbae092-B15] Mangul S , Martin LS, Langmead B et al How bioinformatics and open data can boost basic science in countries and universities with limited resources. Nat Biotechnol 2019;37:324–6. 30833765 10.1038/s41587-019-0053-y

[vbae092-B16] Metsalu T , Vilo J. ClustVis: a web tool for visualizing clustering of multivariate data using principal component analysis and heatmap. Nucleic Acids Res 2015;43:W566–70. 25969447 10.1093/nar/gkv468 PMC4489295

[vbae092-B17] Robinson MD , McCarthy DJ, Smyth GK. edgeR: a Bioconductor package for differential expression analysis of digital gene expression data. Bioinformatics 2009;26:139–40. 19910308 10.1093/bioinformatics/btp616 PMC2796818

[vbae092-B18] Tang Z , Kang B, Li C et al GEPIA2: an enhanced web server for large-scale expression profiling and interactive analysis. Nucleic Acids Res 2019;47:W556–60. 31114875 10.1093/nar/gkz430 PMC6602440

[vbae092-B19] Thorsson V , Gibbs DL, Brown SD et al The immune landscape of cancer. Immunity 2018;48:812–30.e814. 29628290 10.1016/j.immuni.2018.03.023 PMC5982584

[vbae092-B20] Tolaney SM , Garrett-Mayer E, White J et al Updated standardized definitions for efficacy end points (STEEP) in adjuvant breast cancer clinical trials: STEEP version 2.0. J Clin Oncol 2021;39:2720–31. 34003702 10.1200/JCO.20.03613 PMC10166345

