# Supplementary material for: Evergene: an interactive webtool for large-scale gene-centric analysis of primary tumours
Source: Bioinform Adv. 2024 Jun 18;4(1):vbae092. doi: 10.1093/bioadv/vbae092 (PMC11213629; doi:10.1093/bioadv/vbae092)
Supplement: vbae092_Supplementary_Data [file vbae092_supplementary_data.zip › Supplementary_Table3_tool_comparison.docx]

| Webtool | Specify clinical event | Specify high/low expressing groups | Visualise spread of low/high gene expression | Compare gene expression to clinical events | Multiple input gene | Gene set analysis | Multiple plot and plot data export |
| --- | --- | --- | --- | --- | --- | --- | --- |
| Evergene | Up to 4 options | Yes | Yes | Yes | Yes | No | Yes |
| Survival Genie | 2 options | Yes | No | No | (Yes, but 1 output as a gene set) | Yes | No |
| Kaplan-Meier plotter | Up to 4 options | (auto cutoff or pre-set grouping) | No | No | Yes | No | (multiple plots; individual export) |
| GEPIA2 | 2 options | Yes | No | No | (Yes, but 1 output as a gene set) | Yes | No |

**Supplementary Table 3.** Comparison between Evergene and existing webtools for survival analysis.
