# Supplementary material for: Evergene: an interactive webtool for large-scale gene-centric analysis of primary tumours
Source: Bioinform Adv. 2024 Jun 18;4(1):vbae092. doi: 10.1093/bioadv/vbae092 (PMC11213629; doi:10.1093/bioadv/vbae092)
Supplement: vbae092_Supplementary_Data [file vbae092_supplementary_data.zip › Figure S1.pdf]

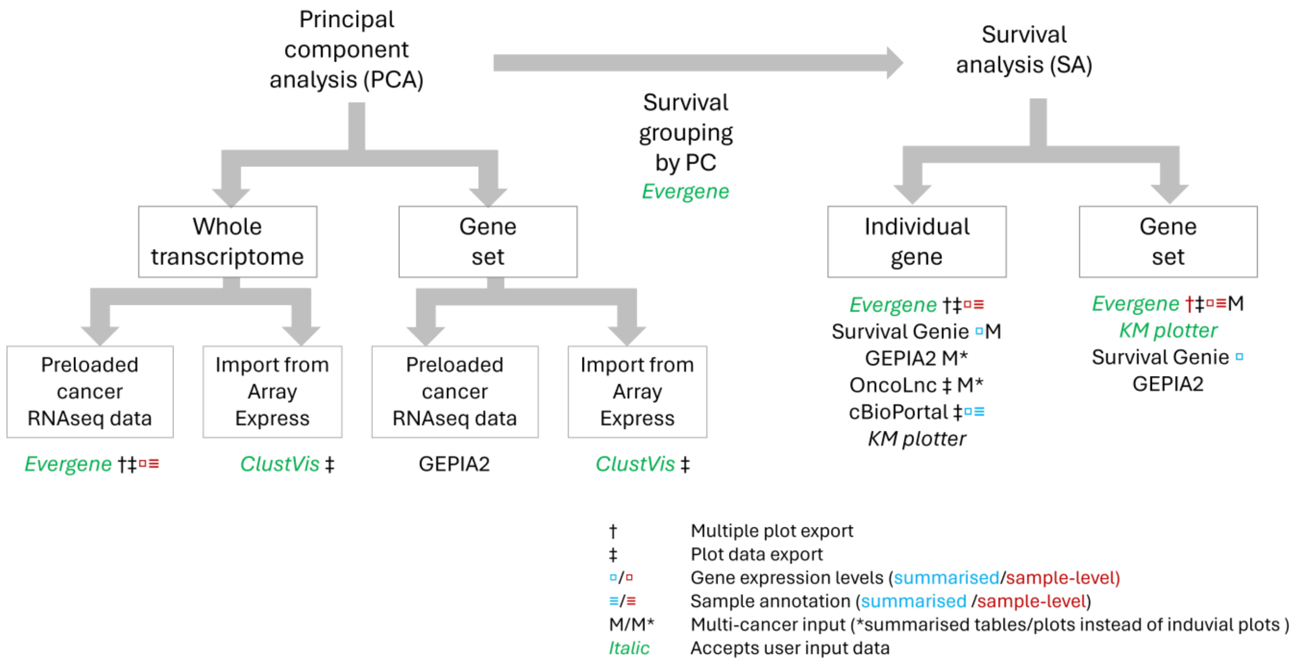

Figure S1. Comparison of existing webtools for principal component analysis (PCA), and survival analysis (SA) or correlation analysis (CA) on their genes of interest. As well as being the only tool that can be used to perform PCA on whole transcriptome using preloaded cancer RNAseq data, Evergene is the only tool that supports using PCs from PCA to perform grouping in SA. Furthermore, Evergene has a comprehensive set of functionality that supports large-scale exploration with multiple genes/cancers.
