# Supplementary material for: Evergene: an interactive webtool for large-scale gene-centric analysis of primary tumours
Source: Bioinform Adv. 2024 Jun 18;4(1):vbae092. doi: 10.1093/bioadv/vbae092 (PMC11213629; doi:10.1093/bioadv/vbae092)
Supplement: vbae092_Supplementary_Data [file vbae092_supplementary_data.zip › Figure S2.pdf]

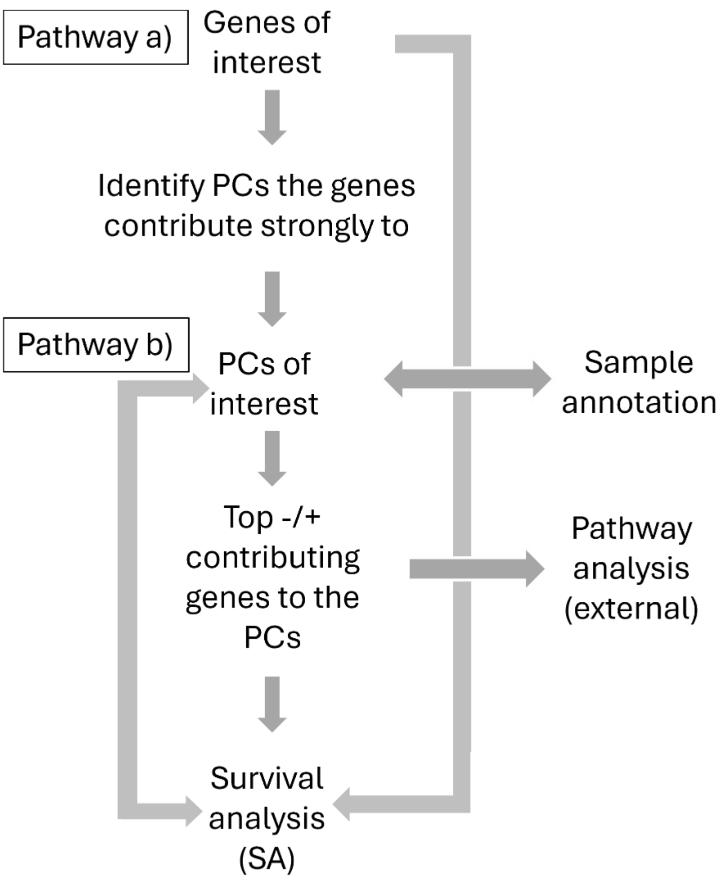

Figure S2. Example workflow.

Users can use Evergene with workflows that centred around specific genes of interest (Pathway a) or without prior knowledge other than the cancers of interest (Pathway b). **a)** When there are specific genes of interests, users may wish to use principal component analysis (PCA) to identify principal components (PCs) the genes contribute strongly, or utilise SA to determine if the expression of their genes of interest or gene set corresponds to survival outcomes. Upon identifying PCs of interest, the users can then compare the PCA plot against known sample annotation to determine if the spread of the data corresponds to known sample characteristics. **b)** Alternatively, users without prior genes of interest can leave the input gene list blank and explore the PCs against sample annotation to determine a specific PC of interest. Once the PCs of interest have been identified through sample annotation or survival analysis, users can use genes that contribute strongly positively (+) or negatively (-) to these PCs for further analysis, such as SA and correlation analysis within Evergene, or pathway analysis using external tools after downloading the top 100 genes for each PC from Evergene.
